# A Novel Case of *IFNAR1* Deficiency Identified a Common Canonical Splice Site Variant in *DOCK8* in Western Polynesia: The Importance of Validating Variants of Unknown Significance in Under-Represented Ancestries

**DOI:** 10.1007/s10875-024-01774-x

**Published:** 2024-08-05

**Authors:** Aimee Huynh, Paul E Gray, Anna Sullivan, Joseph Mackie, Antoine Guerin, Geetha Rao, Karrnan Pathmanandavel, Erika Della Mina, Georgina Hollway, Matthew Hobbs, Karen Enthoven, Patrick O’Young, Sam McManus, Luke H. Wainwright, Megan Higgins, Fallon Noon, Melanie Wong, Paul Bastard, Qian Zhang, Jean-Laurent Casanova, Kuang-Chih Hsiao, Alberto Pinzon-Charry, Cindy S Ma, Stuart G. Tangye

**Affiliations:** 1 Queensland Paediatric Immunology and Allergy Service, Children’s Health Queensland, Brisbane, QLD 4101 Australia; 2 Clinical Immunogenomics Research Consortium, Australasia, Australia; 3 School Medicine, Western Sydney University, Penrith, NSW Australia; 4 Department of Immunology and Infectious Diseases, Sydney Children’s Hospital, Randwick, Australia; 5 Garvan Institute of Medical Research, 384 Victoria St, Darlinghurst, NSW 2010 Australia; 6 School of Clinical Medicine, Faculty of Medicine and Health, UNSW Sydney, Sydney, NSW Australia; 7 Royal Brisbane and Women’s Hospital, Herston, QLD Australia; 8 Genetic Health Queensland, Brisbane, Australia; 9 Department of Allergy and Immunology, The Children’s Hospital at Westmead, Sydney, NSW Australia; 10 Laboratory of Human Genetics of Infectious Diseases, Necker Branch, INSERM U1163, Necker Hospital for Sick Children, Paris, France; 11 St. Giles Laboratory of Human Genetics of Infectious Diseases, Rockefeller Branch, The Rockefeller University, New York, NY USA; 12 University of Paris, Imagine Institute, Paris, France; 13 Department of Pediatrics, Necker Hospital for Sick Children, AP-HP, Paris, France; 14 Howard Hughes Medical Institute, New York, USA; 15 Starship Child Health, Auckland, New Zealand; 16 Department of Paediatrics, University of Auckland, Auckland, New Zealand; 17 Griffith University and University of Queensland, Queensland, Australia

## Abstract

Advanced genomic technologies such as whole exome or whole genome sequencing have improved diagnoses and disease outcomes for individuals with genetic diseases. Yet, variants of unknown significance (VUS) require rigorous validation to establish disease causality or modification, or to exclude them from further analysis. Here, we describe a young individual of Polynesian ancestry who in the first 13 mo of life presented with SARS-CoV-2 pneumonia, severe enterovirus meningitis and adenovirus gastroenteritis, and severe adverse reaction to MMR vaccination. Genomic analysis identified a previously reported pathogenic homozygous variant in *IFNAR1* (c.1156G > T, p.Glu386* LOF), which is common in Western Polynesia. Moreover, a new and putatively deleterious canonical splice site variant in *DOCK8* was also found in homozygosity (c.3234 + 2T > C). This *DOCK8* variant is common in Polynesians and other under-represented ancestries in large genomic databases. Despite *in silico* bioinformatic predictions, extensive in vitro and ex vivo analysis revealed the *DOCK8* variant likely be neutral. Thus, our study reports a novel case of IFNAR1 deficiency, but also highlights the importance of functional validation of VUS, including those predicted to be deleterious, and the pressing need to expand our knowledge of the genomic architecture and landscape of under-represented populations and ancestries.

## Introduction

IFNαR1-deficiency (IFNAR1, OMIM #107450), due to biallelic damaging variants in *IFNAR1*, is an inborn error of immunity underlying susceptibility to severe disease due to live attenuated viruses in measles-mumps-rubella/varicella (MMR/V) or yellow fever vaccines [1–3], and natural infection with viruses including influenza, herpes simplex and SARS-CoV-2 [3–8]. These infections are also seen in individuals with autosomal recessive *IFNAR2* deficiency [9, 10], or autoAbs neutralizing type I IFNs (IFNα, IFNω) [8, 11–13]. To date, fewer than 20 individuals with biallelic variants in *IFNAR1* have been reported [3, 14]. Notably, we recently identified 7 of these patients from 5 unrelated families of Western Polynesian ancestry in the Pacific region [15]. All patients were homozygous for the *IFNAR1* c.1156G > T, p.Glu386* loss-of function (LOF) variant, resulting in severe live attenuated viral vaccine (LAV)-associated disease in 6/7 cases, being fatal in 4 cases. Strikingly, despite being exceedingly rare in commonly-used genome reference databases (1 out of 1504192 alleles on gnomAD 4.0) [16], the *IFNAR1* p.Glu386* LOF variant was found to be common in individuals of Western Polynesian ancestry, with an estimated minor allele frequency of > 1% [15]. Homozygosity for this variant abolishes responses of patient fibroblasts to type I IFNs [15]. Delays in diagnosis of IFNαR deficiency can lead to life-threatening viral infections with frequently fatal outcomes [3, 6, 14]. There are no standard prophylaxis or treatment guidelines for IFNAR1-deficient patients [3]. However, due to the innate immune defects, acute LAV-associated hyperinflammatory disease has been treated with immune modulators including steroids and high dose intravenous immunoglobulin (IVIg), while empirical prophylaxis has included the use of pooled polyclonal IgG infusions, antiviral prophylaxis, avoidance of live viral vaccines, and behavioral changes to avoid infection with endemic or epidemic viruses. Even though penetrance and expressivity of IFNAR1 deficiency can be variable [3, 6, 7], the significant mortality and burden of disease in the Pacific Islands mandates for increased awareness, and improved understanding of the role of type I IFNs in viral clearance to refine treatment recommendations and improve outcomes for these patients.

The full range of clinical consequences of IFNAR1 deficiency remains unclear, especially in patients from Western Polynesia. Moreover, there may be alleles in other known disease-causing genes that are rare world-wide but common in Polynesia. Some Polynesians may thus have two recessive inborn errors of immunity. Alternatively or additionally, the clinical phenotype of IFNAR1 deficiency may be modified by another recessive inborn error. This was tested in our current study by the identification of homozygous variants in *IFNAR1* and *DOCK8* in a young individual presenting with a history of severe and recurrent viral infections.

## Methods

### Ethics Statement

Buffy coats were purchased from the Australian Red Cross Blood Service. Peripheral blood was collected from the affect patient, as well as additional patients with confirmed variants in *IFNAR1* [15] or *DOCK8*. This study was approved by the Sydney Local Health District RPAH Zone Human Research Ethics Committee and Research Governance Office, Royal Prince Alfred Hospital, Camperdown, NSW, Australia (Protocols X16-0210/LNR/16/RPAH/257 and X16-0210 & 2019/ETH06359); and St Vincent’s Hospital (Darlinghurst, NSW, Australia) Human Research Ethics Committee (Protocol X20-0177 & 2020/ETH00998). Written informed consent was obtained from participants or their guardians. Experiments using samples from human subjects were conducted in accordance with local regulations and with the approval of the IRBs of corresponding institutions.

### STAT1 Phosphorylation

T cell blasts were established by stimulating total PBMCs with ImmunoCult Human CD2/CD3/CD28T cell activator with IL-2 (0.02ng/ul, StemCell, cat #78036) in ImmunoCult-XF T Cell Expansion Medium (StemCell, cat #10981). After 14 days of expansion, cells were rested in RPMI overnight and then stimulated in media alone or in the presence of IFNβ (100ng/mL) or IL-21 (100ng/mL) for 30 min. Cells were then harvested, fixed, permeabilised and stained with conjugated anti-pSTAT1 PE mAbs (4 A, BD Phosflow, cat #612564) and analysed on the BDFortessa flow cytometer [17].

### Analysis of DOCK8 Expression

#### FACS

PBMCs from healthy donors, patients with pathogenic homozygous loss-of expression variants in *DOCK8* (c.2142G > A, p.W714*), or P1 were incubated with fluorophore-conjugated mAbs against CD3, CD4, and CD8, and then fixed, permeabilized, and stained with anti-DOCK8 mAb, as previously described [18–21].

#### Western Blot

Total protein extracts were prepared from T-blast generated from healthy donors, a DOCK8-deficient patient or P1 by mixing cells total cell lysis buffer (50 mM Tris-HCl pH 7.4, 150 mM NaCl, 0.5% Triton X-100, and 2 mM EDTA) supplemented with protease inhibitors (Complete Mini Protease Inhibitor Cocktail, #4693124001, Roche), a phosphatase inhibitor cocktail (PhoStop, # #4906837001, Roche), and 0.1 mM dithiothreitol (DTT, Thermo Fisher). Lysis was performed on ice for 40 min (hard vortex every 10 min). Equal amounts of protein (Bradford protein assay #5000006, Bio-Rad) were resolved by SDS-PAGE using 4–20% Criterion TGX precast polyacrylamide gels (#5671093, Bio-Rad) and transferred to a Immun-Blot Low Fluorescence PVDF Membrane (#162–0263, Bio-Rad). Membranes were probed with antibodies against DOCK8 (unconjugated, clone EPR12511, #ab175208, Abcam) or GAPDH (unconjugated, clone 6C5, #sc-32233, Santa Cruz). Primary antibodies were detected by incubation of membranes with donkey anti-rabbit IRDye 800CW (#926-32213, Licor) and donkey anti-mouse IRDye 800CW (#926-32212, Licor). Binding was detected with Odyssey CLx Imager (Licor). The Chameleon Duo Prestained Protein Ladder (#928-60000, Licor) was used to provide molecular weight marker. Images were analyzed with Image studio software (Licor).

### *DOCK8* PCR Amplification and Sequencing

RNA was extracted using the Zymo Quick-RNA microprep kit (#R1051, Zymo); residual genomic DNA was removed using Zymo Spin-Away filters (#C10006-250-F). RNA was reversed-transcribed with the High-Capacity RNA-to-cDNA Kit (#4387406, Applied Biosystems), according to the manufacturer’s protocol. *DOCK8* cDNA amplification was performed on cDNA extracted from PBMCs from healthy donors or P1 using the following primers targeting exon 23 (5’-TCTGGCAGTAGTGATGCT-3’) and exon 29 (5’-TGGTTTGACACAGCGT-3’), MyTaq DNA polymerase (#BIO-21105, Bioline) and the following conditions: initial denaturation (95 °C, 1 min) followed by 30 cycles of denaturation (95 °C, 1 min), annealing (60 °C, 15 s), extension (72 °C, 10s) followed by a final extension step (72 °C, 2 min). *DOCK8* amplicons were separated by 1% agarose electrophoresis. The different amplicons were then purified with Monarch DNA Gel Extraction kit (#T1020L, New England Biolabs) and sequenced by the Sanger sequencing method.

### Immunophenotyping

PBMCs from healthy donors, patients with confirmed pathogenic biallelic variants in *DOCK8* or *IFNAR1*, or P1 were incubated with fluorophore-conjugated mAbs against CD3, CD4, CD8, CD45RA, CCR7 and CD57. Percentages of: total (CD3^+^), CD4^+^ (CD3^+^CD4^+^) and CD8^+^ (CD3^+^CD8^+^) T cells; naive (N; CD45RA^+^CCR7^+^), central memory (T_CM_; CD45RA^−^CCR7^+^), effector memory (T_EM_; CD45RA^−^CCR7^−^), and CD45RA^+^ revertant memory (T_EMRA_; CD45RA^+^CCR7^−^) cells amongst CD4^+^ and CD8^+^ T cells; and naive, T_CM_, T_EM_ and T_EMRA_ CD8^+^ T cells expressing CD57 were determined, as previously described [18–22].

### Lymphocyte Isolation and Functional Analysis

Memory CD4^+^ T cells (CD3^+^CD4^+^CD45RA^−^) were isolated by cell sorting from PBMCs of healthy donors, a DOCK8-deficient patient, or P1. These cells were then cultured under Th0 conditions for 5 days, after which time secretion of Th2 (IL-4, IL-5, IL-13) and Th17 (IL-17 A, IL-17 F, IL-22) cytokines were determined by cytometric bead arrays (BD) or ELISA as previously described [18–20].

## Results

### Clinical History

**Fig. 1:**
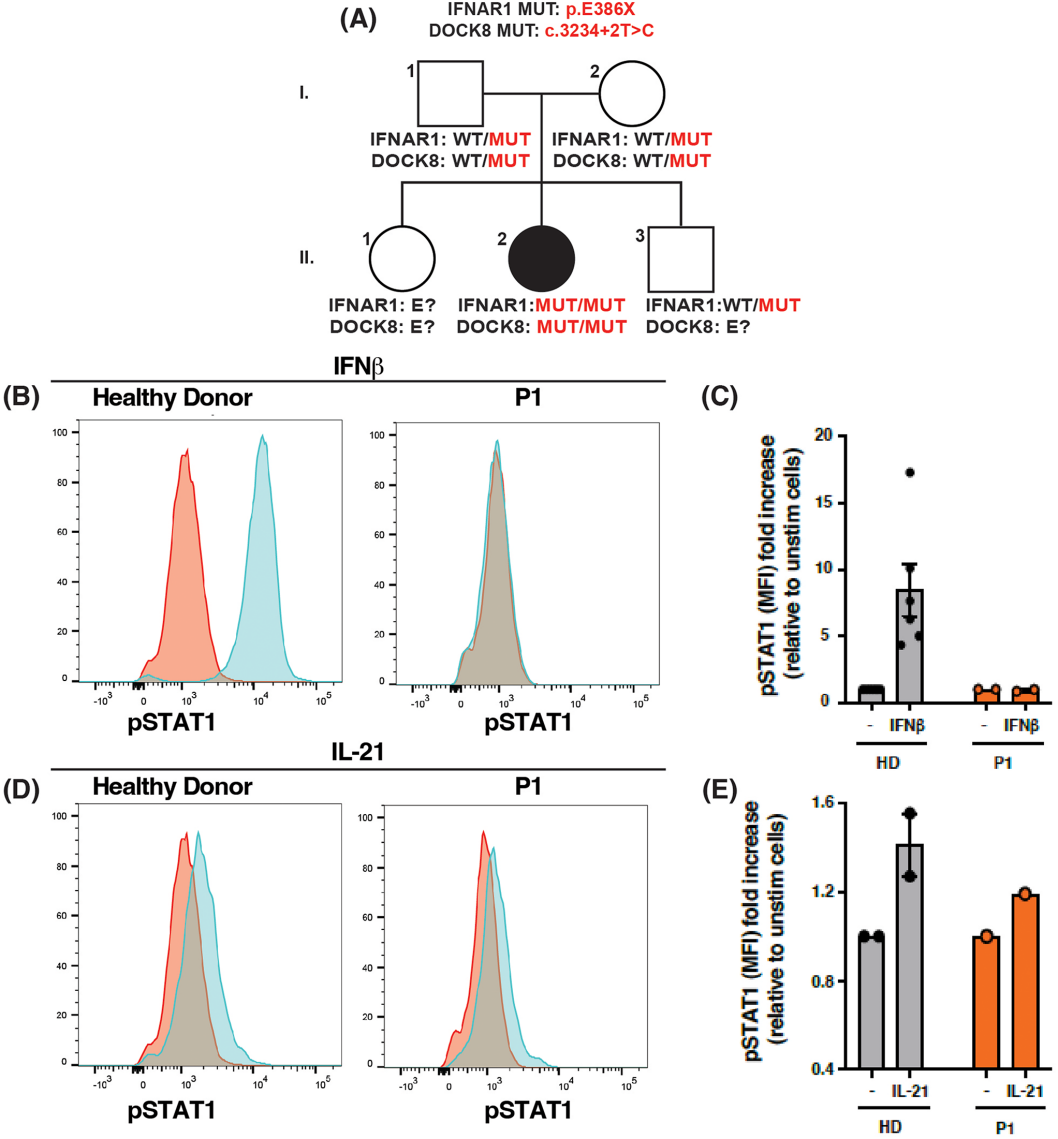
*IFNAR1* (c.1156G > T, p.(Glu386*) variant is loss-of function in T cells (**A**) Pedigree of a novel IFNAR1-deficient kindred. Familial segregation of the *IFNAR1* c.1156G > T, p.E386X and *DOCK8* c.3234 + 2T > C variants are shown. The affected individual P1 is represented by a black symbol (II.2). Individuals of unknown genotype are labelled “E?”. (**B**-**E**) T cell blasts were expanded from healthy donors (HD) and P1 by stimulating PBMCs with anti-CD2/CD3/CD28 mAbs. After 14 days, the cells were rested, and then stimulated in the absence or presence of (**B**, **C**) IFNb (type I IFN) or (**D**, **E**) IL-21. After 15 min, cells were harvested, fixed and permeabilised and then stained with mAb specific for phospho-STAT1. FACS plot (**B**, **C**) are representative of pSTAT1 induction in T-cell blasts from HD or P1. The data in (**C**) and (**E**) represent the fold-change in pSTAT1 gMFI in T-cell blasts stimulated with IFNβ or IL-21, respectively, relative to unstimulated cells. Each point represents data from an independent experiment

A 15-month-old female (P1) born to non-consanguineous parents of Samoan origin (Fig. 1A, II.2) was noted in the first few months of life to have developmental delay and clinical dysmorphology including arthrogryposis. During the first 12 months of life, she had a significant history of severe and repeated respiratory and gastrointestinal illnesses. At 4 months of age, she had severe hypoxemic SARS-CoV2-induced pneumonia that required supplemental oxygen and was treated with high dose intravenous dexamethasone as per institutional guidelines. No passive SARS-CoV2 immunity was expected as maternal vaccination was commenced 2 months after the patient’s birth. At 6 months, she had severe enterovirus meningitis and gastroenteritis requiring prolonged hospital admission for fluid and feeding support; at 13 months she had severe adenovirus gastroenteritis requiring electrolyte and fluid therapy in hospital. Finally, 10 days after receiving MMR vaccination she became very unwell, presenting with protracted lethargy, irritability, intermittent fevers, rash, tachypnoea, anaemia, thrombocytopaenia, splenomegaly, hyperferritinaemia, hypofibrinogenemia and rapidly progressive hepatitis (Table 1). Clinical examination did not revealed any evidence of atopic disease, nor is there any evidence of eczema, or allergies to food or environmental allergens.

Prompt immunological evaluation to investigate possible primary hemophagocytic lymphohistiocytosis (HLH) was undertaken. NK cell degranulation (CD107a expression), and assessment of intracellular perforin, XIAP, SAP and surface HLA-DR expression by flow cytometric assays were within laboratory reference ranges of healthy donors. While soluble CD25 and ferritin levels were elevated, fibrinogen levels were reduced (Table 1) and a bone marrow aspirate did not show evidence of hemophagocytosis. Further immune work-up revealed panlymphopenia and hypergammaglobulinemia (Table 1). Overall, P1 developed a hyperinflammatory state that included some features of HLH. Interestingly, HLH was recently described in an unrelated case of IFNAR1 deficiency [2].

**Table 1:** Cellular, serological, inflammatory and infectious features of P1

Immune parameter	P1	Reference range
Total lymphocytes	1.07 × 10^9^/ml (ii)	3.5–11 × 10^9^/ml
CD3 + T cells	0.55 × 10^9^/ml (ii)	1.6–6.7 × 10^9^/ml
CD4 + T cells	0.24 × 10^9^/ml (ii)	1.6–6.7 × 10^9^/ml
CD8^+^ T cells	0.32 × 10^9^/mL (i)	0.4–2.1 × 10^9^/ml
B cells	0.4 × 10^9^/mL (ii)	0.6–2.7 × 10^9^/ml
NK cells	0.06 × 10^9^/mL (ii)	0.2–1.2 × 10^9^/ml
IgG	17.2 g/L (hh)	3.0–13.0 g/L
IgM	3.3 g/L (h)	0.5–1.6 g/L
IgA	1.2 g/L (in range)	0.1–1.4 g/L
IgE	9 kU/L (in range)	< 100 kU/L
ferritin	1480 µg/L (hh)	5–100 µg/L
Soluble CD25	23 601 pg/mL (hh)	< 7887 pg/mL
Fibrinogen	0.4 g/L (ii)	2–4.5 g/L
**CSF cytology/chemistry**		
Glucose	3 mmol/L (i)	3.3–4.5 mmol/L
Protein	210 mg/L	150–500 mg/L
WCC	6 × 10^6^/L (h)	0–5 × 10 × ^6^/L
Viral PCR	Negative for: adenovirus, enterovirus, parechovirus, HSV1/HSV2, CMV, measles, JC virus	NA

Extensive metabolic and malignant screens were also negative, while MRI brain, and chest/abdomen/pelvis CTs were normal. Furthermore, an extended infectious screen confirmed measles vaccine strain in the urine, and mumps and rubella viruses – but not measles - in nasopharyngeal and respiratory secretions. To exclude neuroinflammation and/or infection, CSF analysis was performed. Cytology and biochemistry were normal. Extensive CSF cultures and viral PCRs were negative, including for adenovirus, CMV, enterovirus, HSV1/2, parechovirus, JCV, WT and vaccine measles). Measles IgG was also non-reactive in CSF (Table 1). The patient has made a full recovery (current age: 30 months) and remains on Ig replacement therapy and regular surveillance. P1 has 2 healthy siblings, currently aged 4 yrs (II.1) and 4 months (II.3, Fig. 1A).

### Outcomes of Genetic Testing

Based on clinical phenotype of LAV-associated disease with hyperinflammatory complications, and the ethnicity of the patient and parents, IFNAR1 deficiency was suspected. Urgent whole genome sequencing was performed on whole blood DNA from the patient, with initial analysis focussed on genes known to cause metabolic, immunological, aortopathy and connective tissue disorders, as well as congenital hypothyroidism, and arthrogryposis. This identified three clinically relevant variants in P1:

 [1] a pathogenic homozygous nonsense variant in *IFNAR1* (c.1156G > T, p.Glu386* LOF) as previously described in patients from the Pacific Islands [15];

 [2] a likely pathogenic heterozygous variant in *FBN2* (c.4346-1G > C, p.?), and.

 [3] a homozygous variant of uncertain significance (VUS) in *DOCK8* (NM_203447.4, c. 3234 + 2T > C) predicted to abolish the highly conserved + 2 donor splice site (phyloP score 7.85 [-19.0-10.9]), phastCons score: 1 [0, 1]; http://compgen.cshl.edu/phast/) at the intron 26/exon 26 junction (Fig. 1A). As expected, genotyping each parent (I.1, I.2) revealed them to be heterozygous for the variants in *IFNAR1* (c.1156G > T, p.Glu386* LOF) and *DOCK8* (c. 3234 + 2T > C) (Fig. 1A).

Heterozygous pathogenic variants in *FBN2* have been associated with congenital contractual arachnodactyly [23]. The *FBN2* variant idenitifed in P1 was absent from gnomAD and has not been previously reported. It was considered to be pathogenic as parental segregation revealed it to be de novo in P1; this private *FBN2* variant confirmed a diagnosis of congenital contractual arachnodactyly in P1. Thus, the *IFNAR1* and *DOCK8* variants were therefore investigated in more detail.

### The *IFNAR1* Glu386*Variant is LOF

The *IFNAR1* (c.1156G > T, p.(Glu386*) variant identified in P1 has previously been shown to be LOF in over-expression systems and functional analysis in fibroblasts generated from an affected individual [15]. As the functional requirements of type I IFN signalling in host defence can differ between immune and non-immune cells [3, 7, 13], it was important to establish the *IFNAR1* Q386* variant was also deleterious in leukocytes. To test this, T cell blasts were generated from PBMCs from P1 as well as healthy donors (HD), and then assessed for induction of STAT1 phosphorylation in response to IFNβ. In the presence of IFNβ, STAT1 phosphorylation was increased 5-10-fold in T-cell blasts from HD compared to unstimulated cells (Fig. 1B left panel, Fig. 1C). In contrast, no pSTAT1 could be detected in IFNβ-stimulated T-cell blasts from P1 (Fig. 1B right panel, Fig. 1C). Importantly, induction of pSTAT1 in T-cell blasts from HD and P1 stimulated with IL-21 was intact (Fig. 1D, E), establishing that the defect in response to IFNβ in P1 T cells was specific and not a result of generalised impairment of cytokine signalling. Thus, similar to our previously reported patients of Western Polynesian ancestry [15], *IFNAR1* was LOF in P1.

### *DOCK8* 3234 + 2T > C – In Silico Characteristics and Population Genetics

**Fig. 2:**
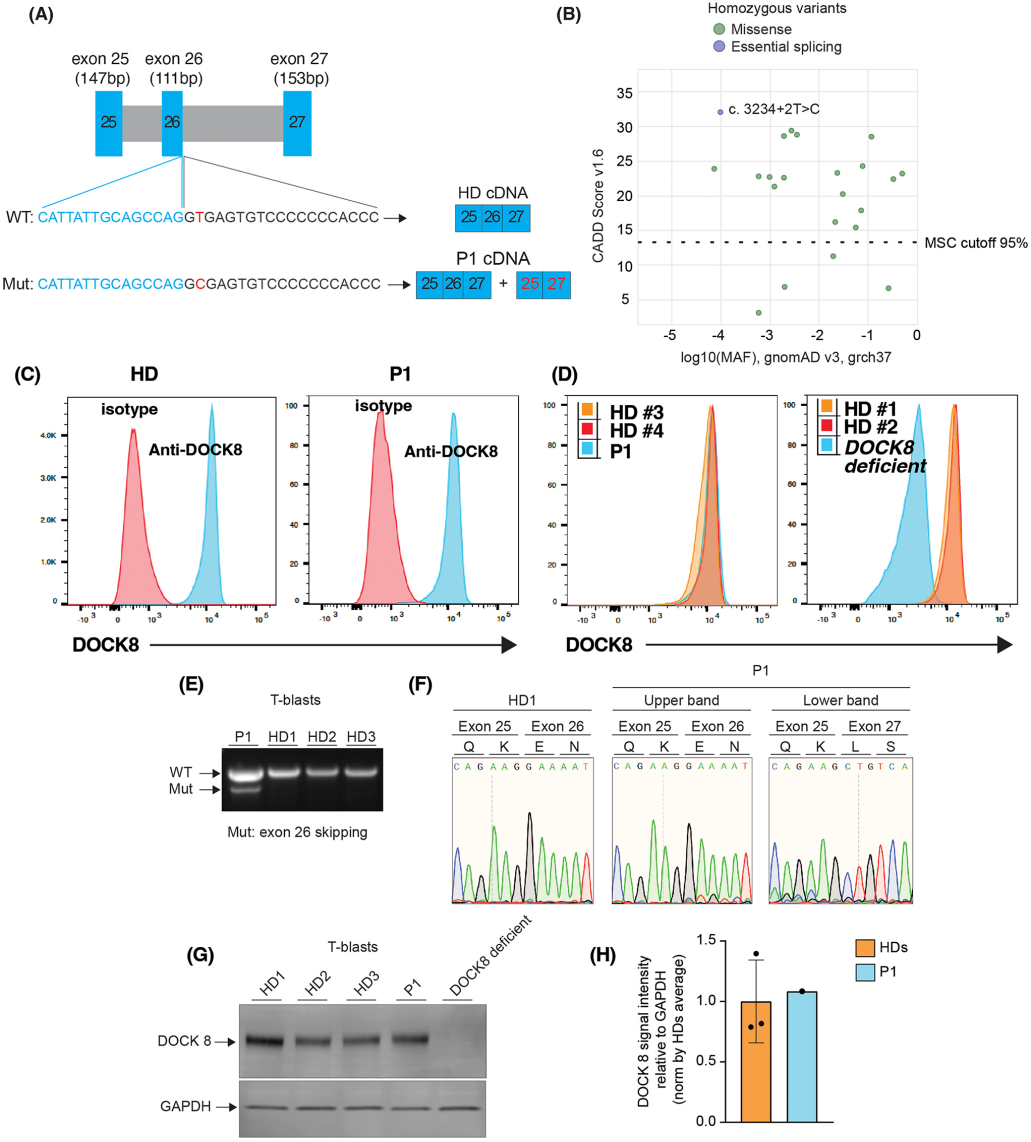
DOCK8 expression is intact in lymphocytes from the IFNAR1-deficient patient harbouring the *DOCK8* c.3234 + 2T > C variant (**A**) schematic of *DOCK8* genomic sequence representing the 5’ region of exon 26 (blue), the 3’ region of intron 26 (black) and the variant found in P1 (red). Prediction of the cDNA sequence is reported for both WT *DOCK8* and *DOCK8* c.3234 + 2T > C. (**B**) Minor allele frequency (MAF, x-axis) and CADD scores (y-axis) for missense (green circles) and essential splicing (purple circles) variants in *DOCK8* reported as homozygous in the public database gnomAD (GRCh37). The *DOCK8* c.3234 + 2T > C variant found in P1 (indigo circles) is the only homozygous essential splicing variant previously reported in gnomAD. The mutation significance cut-off (MSC, 95% confidence interval, y axis) is represented by the dotted line. (**C**, **D**) PBMCs from healthy donors (HD), P1 and a confirmed DOCK8-deficient patient were fixed and permeabilised and stained with anti-DOCK8 mAb. Intracellular expression of DOCK8 was then determined. (**A**) DOCK8 expression (blue histogram) in PBMCs from a HD (left) and IFNAR1-deficient patient P1 (right) relative to staining with isotype IgG control mAb. (**B**) Overlay of DOCK8 expression in PBMCs from HDs (#1-#4), P1 (left) or a DOCK8-deficient patient (right). (**E**, **F**) exons 25–27 of *DOCK8* were PCR amplified from PBMCs of healthy donors and P1. (**E**) agarose gel depicting amplified products, including 2 products for P1. (**F**) Sequencing of amplified PCR products from HD (left) and P1 (right) showing the higher molecular weight band from P1 corresponds to WT *DOCK8* cDNA, while the lower band lacks exon 26. (**G**, **H**) whole cell lysates were prepared from T blasts expanded from healthy donors (HD), P1 and a confirmed DOCK8-deficient patient. DOCK8 expression was assessed by SDS-PAGE and Western blotting. Detection of GADPH was used as a loading control. (**G**) representative of western blot from 3 different experiments. (**H**) summary data depicting expression of DOCK8 protein in lysates from 3 unrelated healthy donors and P1, relative to GAPDH

The homozygous *DOCK8* variant (NM_203447.4, c. 3234 + 2 T > C) in P1 is localized in intron 26, 2 bp after the exon 26 splicing donor site (Fig. 2A). The T > C substitution is reported to be likely pathogenic in dbSNP database (https://www.ncbi.nlm.nih.gov/snp/, rs756871628) and ClinVar (https://preview.ncbi.nlm.nih.gov/clinvar/, 573664). *In silico* analysis using multiple programs (MaxEntScan, NNSplice, GeneSplicer) predicted the variant will have an adverse effect on the splicing donor site, potentially leading to abolition of splicing and skipping of exon 26 (Fig. 2A). However, SpliceSiteFinder-like program predicted the variant would only decrease the donor site usage by 7.5% (WT score: 95.6%; variant score: 88.5%) and thus have only a modest impact on splicing. The variant has a combined annotation-dependent depletion (CADD) score of 32, well above the mutation significance cutoff (MSC) for *DOCK8* (13.3) (Fig. 2B) [24, 25].

Interestingly, the variant appears common in certain populations. Specifically, 28 heterozygous individual and one homozygous individual are present in gnomAD (v4.0.0), with 25/29 individuals being either Admixed American (*n* = 10), or ‘remaining’ (*n* = 15) ethnicity. There were no homozygotes for any other predicted LOF variants in *DOCK8* in any public database. The *DOCK8* c.3234 + 2T > C variant was not found in 2844 healthy elderly adults from the Medical Genome Reference Bank (MGRB) [26]. The ethnicity of the majority of the MGRB is non-Finnish European, with no known Polynesian representation. In terms of available population frequencies, the *DOCK8* c.3234 + 2T > C variant has a MAF of 0.108 in Vanuatu in the Western part of Western Polynesia [27], but there is a lack of published data for other populations. In terms of variant distribution however, retrospective analysis of genomic data from > 25 000 patients who had undergone gene panel/WES/WGS for the diagnosis of various conditions found this variant in a heterozygous state in one individual from the Philippines, as well as 12 individuals of Polynesian ancestry (most residing in Australia or Aotearoa New Zealand) including one of the previously-reported patients with the homozygous *IFNAR1* c.1156G > T; pGlu386* variant [P3 in reference [15]]. The occurrence in ‘Admixed Americans’ on the gnomAD database may reflect a broader distribution outside of Polynesia, or could potentially reflect ethnicity from, for instance, American Samoa or Hawaii in North Western Polynesia. Thus, similar to the *IFNAR1* c.1156G > T variant, the *DOCK8* c.3234 + 2T > C variant appears to have a high MAF in Polynesians and ‘Admixed Americans’.

### Effect of the Homozygous *DOCK8* Variant on Protein Expression

While the clinical phenotype of P1 was not strictly suggestive of DOCK8 deficiency, she was only 15 months old and clinical onset of DOCK8 deficiency can be variable within the first 5 years of life [28–30]. Furthermore, due to a paucity of reports of DOCK8-deficiency in the Pacific regions, it is possible the clinical phenotype of affected individuals in this geographical area may differ from that reported for DOCK8-deficiency in the Americas, Europe and the Middle East [18, 29, 31, 32]. Lastly, as the *DOCK8* variant was homozygous and located in a splice region it was important to assess potential pathogenicity to determine if P1 also had DOCK8 deficiency, as the treatments for IFNAR1-deficiency and DOCK8-deficiency are different [3, 28–30].

To investigate the impact of the *DOCK8* c.3234 + 2T > C variant, we first assessed intracellular expression by flow cytometry [18–21]. Similar levels of DOCK8 were detected in PBMCs from HDs and P1 (Fig. 2C left panel, Fig. 2D). This contrasted a patient diagnosed with DOCK8 deficiency (homozygous c.2142G > A variant, p.W714*), whose leukocytes clearly lacked DOCK8 protein (Fig. 2D [right panel]).

The *DOCK8* c.3234 + 2T > C variant was predicted to cause skipping of exon 26 of *DOCK8*, thus resulting in expression of a truncated protein (Fig. 2A). It was possible that this shorter protein could still be detected by the mAb used for flow cytometry. To explore this further, we performed PCR using primers that would amplify across exons 23–29. This amplified a 798 bp product from cDNA generated from PBMCs of 3 HDs (HD1-3; Fig. 2E). Interestingly, 2 products were amplified from P1 (Fig. 2E): one corresponding to that detected in HDs, and a shorter product lacking exon 26 (114 bp encoding 38 amino acids; Fig. 2E, F).

As this shorter cDNA represented a relatively low proportion of amplified product, it was unlikely to be the dominant protein isoform. Indeed, Western blotting using a DOCK8-specific Ab detected a protein of comparable molecular weight in lysates extracted from T-cell blasts expanded from 3 HDs and P1 (Fig. 2G, H). The absence of detectable DOCK8 protein in lysates from T cells expanded from a known DOCK8-deficient patient confirmed the specificity of the anti-DOCK8 mAb used (Fig. 2G). Thus, consistent with the flow cytometry data, and despite the presence of a truncated cDNA transcript, the *DOCK8* variant detected in P1 does not appear to contribute substantially to expression of total DOCK8 protein.

### T Cells from P1 lack Phenotypic Signatures Features of DOCK8-Deficient T Cells

**Fig. 3:**
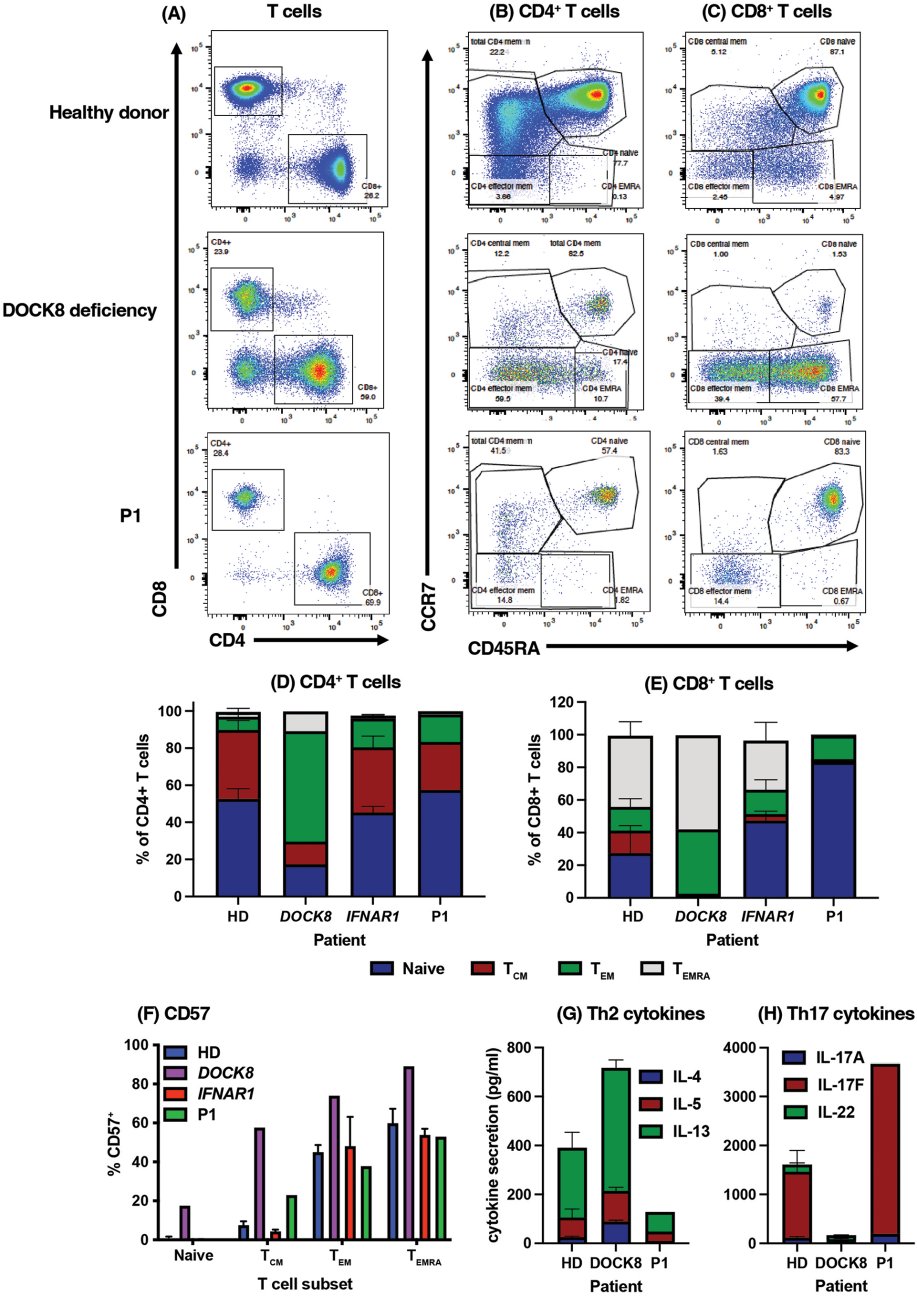
Comparison of the phenotype and function of T cells from patients with IFNAR1-deficiency versus DOCK8-deficiency (**A**-**F**) PBMCs from HDs, one DOCK8 deficient patient, 2 IFNAR1-deficient patients or P1 were stained with mAbs against CD3, CD4, CD8, CD45RA, CCR7 and CD57. (**A**-**C**) representative FACS plots and (**D**-**E**) summary graphs depicting proportions of: (**A**, **D**) CD4^+^ and CD8^+^ T cells within CD3^+^ T cells, (**B**-**E**) naïve, T_CM_, T_EM_ and T_EMRA_ subsets within CD4^+^ (**B**, **D**) and CD8+ (**C**, **E**) T cells in HDs, and the indicated patients. (**F**) expression of CD57 on CD8^+^ naïve, T_CM_, T_EM_ and T_EMRA_ subsets, and the indicated patients. (**G**, **H**) CD45RA^−^ memory CD4^+^ T cells were sorted from healthy donors (HD), a confirmed DOCK8-deficient patient, and P1 and then stimulated in vitro for 5 d with anti-CD2/CD3/CD28 mAb beads. After this time, secretion of (**G**) Th2 cytokines IL-4, IL-5 and IL-13 and (**H**) Th17 cytokines IL-17 A, IL-17 F and IL-22 was determined by cytometric bead array

To provide further evidence that the *DOCK8* c.3234 + 2T > C variant was benign, we assessed the phenotype and function of T cells from P1 for evidence of aberrant DOCK8 function once the acute hyperinflammatory process had resolved. Our previous studies identified a suite of detectable pathognomonic cellular defects in DOCK8-deficient patients, even prior to disease onset [18–22]. Typical DOCK8-deficient patients have an inverted CD4:CD8 ratio (~ 1:2 vs. 2–3:1 for HD), and aberrant CD4^+^ and CD8^+^ T cell compartments (reduced naïve cells/increased T_EM_ and T_EMRA_ cells) compared to HDs [18, 20, 21] (Fig. 3A-C, upper and middle panels; Fig. 3D, E). Similarly, the senescent/exhaustion marker CD57 is expressed on increased proportions of DOCK8-deficient CD8^+^ T cell subsets compared to corresponding subsets in HD [18, 19, 22] (Fig. 3F). When these parameters were determined for P1, we also found a decreased CD4:CD8 ratio (1:2.5, Fig. 3A) which probably reflects hyper-active immunity during convalenscence post-MMR vaccination. Despite this, proportions of naïve and memory CD4^+^ T cell populations, as well as expression of CD57 on CD8^+^ T cell subsets, were comparable to HDs, as well as other patients with the homozygous *IFNAR1* (c.1156G > T, p.(Glu386*) LOF variant (Figure Fig. 3B-F). Furthermore, CD8^+^ T cells in P1 were predominantly naïve, which is in stark contrast to DOCK8-deficiency, even for age-matched patients (Fig. 3B-E).

### Intact Cytokine Production of T Cells from P1

Another characteristic of DOCK8 deficiency is aberrant cytokine production by memory CD4^+^ T cells. Specifically, Th2 cytokines IL-4, IL-5 and IL-13 are dramatically increased in DOCK8-deficient memory CD4^+^ T cells, while that of Th17 cytokines (IL-17A, IL-17F and IL-22) is decreased compared to memory CD4^+^ T cells from HD [18–21]. The cytokine profile was tested by isolating memory CD4^+^ T cells from HDs, a known DOCK8-deficient patient, and P1 and measuring secretion of these cytokines after 5 days of in vitro stimulation. As expected, production of Th2 cytokines was exaggerated for memory CD4^+^ T cells from a confirmed DOCK8-deficient patient compared to HDs, while production of Th17 cytokines from DOCK8-deficient memory CD4^+^ T cells was severely impaired (Fig. 3G, H). Importantly, Th2 cytokine production by P1’s memory CD4^+^ T cells was less than HDs, while Th17 cytokines were intact, greatly exceeding levels produced by DOCK8-deficient memory CD4^+^ T cells (Fig. 3G, H). Combined, our molecular, cellular, and functional studies establish that the *DOCK8* c.3234 + 2T > C variant is benign and highly unlikely to be pathogenic in our patient.

## Discussion

The application of next generation DNA sequencing (NGS) technologies has dramatically increased the rate of molecular diagnoses for many human diseases. This is clear for IEI, where traditional sequencing techniques yielded a diagnosis in ~ 5–10% of cases whereas today a diagnosis is obtained in 30–70% of cases from approaches including targeted sequencing of a large panel of known genes, WES or WGS [33–37]. Whilst this is a terrific advance in molecular medicine, NGS has also posed many challenges, one being the almost-guaranteed identification of novel VUS that cannot easily be classified as neutral or deleterious [38]. This can further delay and complicate achieving a definitive genetic diagnosis, particularly if putative pathogenic variants are identified in > 1 gene known to cause disease. In these cases, it is possible that disease presentation results either from the combined effect of 2 genes [39] or presents as 2 different diseases driven by distinct mechanisms due to pathogenic variants in unrelated genes [40]. Such an outcome can present a therapeutic dilemma because treatment for one IEI may be quite different and even deleterious for another. For these reasons, it is critical that VUS identified by NGS undergo rigorous investigation to determine pathogenicity/disease causality.

In this study, we present the case of a young child with recurrent and severe viral infections who was found to have a heterozygous variant in *FBN2* as well as homozygous variants in both *IFNAR1* and *DOCK8*. While the *FBN2* variant explained congenital contractual arachnodactyly, homozygous variants in *DOCK8* and *IFNAR1* potentially underpinned the infectious complications in this case, thereby necessitating further investigation. Given the significant implication of a potential dual diagnosis, and that standard of care for DOCK8 deficiency is haematopoietic stem cell transplantation (HSCT) [18, 29–31], it was critical to confirm the pathogenicity of the *IFNAR1* variant and investigate the impact of the novel *DOCK8* variant to determine the most appropriate diagnosis and patient management strategy.

The clinical phenotype of both DOCK8 and IFNAR1 deficiencies includes susceptibility to infection with various viruses. This includes herpes viruses (HSV, VZV, EBV), HPV and molluscum for DOCK8-deficiency [29–31], and HSV1, HSV2, SARS-CoV2, influenza and live attenuated viral vaccines for IFNAR1-deficiency [3, 6, 7, 14]. The clinical history of the patient reported here – including SARS-CoV2 infection requiring hospitalisation and supplemental oxygenation, and serious outcomes following MMR vaccination – is consistent with the infectious spectrum of previously-described cases of individuals with IEI that disrupt production of (variants in *IRF7*, *TLR7*) or cellular responsiveness to (variants in eg *IFNAR1*, *IFNAR2*, *STAT2*) type I IFN [3, 6, 41, 42], or with autoAbs neutralizing type 1 IFN [11–13]. However, the potential contribution of the homozygous *DOCK8* variant to disease pathogenesis could not be overlooked. Our assessment of DOCK8 expression and immune cell function in this patient established that this variant is likely neutral, despite strong *in silico* predictions of potential pathogenicity due to its impact on exon splicing. This is consistent with the well-described but clinically rare instances where canonical 5’ splice site GT > GC variants allow production of sufficient wild-type transcript to rescue the phenotype [43]. Indeed, it was recently found that ~ 25% of rare variants that affect canonical splice sites and are predicted to be LOF, and thus pathogenic, had no effect on transcription of the putatively impacted gene [44]. There are several key lessons from our findings:

1. The importance of testing VUS on protein function and biology. By excluding DOCK8-deficiency as a cause of disease, it was possible to proceed with the most appropriate treatment to manage IFNAR1 deficiency, and not consider the need for HSCT.
2. Similar to the *IFNAR1* c.1156G > T, p.Glu386* variant, the *DOCK8* c.3234 + 2T > C variant is extremely rare in publicly available genome/exome databases (MAF: 1.87 × 10^− 5^), but appears to be common in some Oceania and potentially admixed American ancestries, which further underscores the benign nature of this variant. Indeed, this variant has a MAF of 0.108 in people from Vanuatu [27], and was detected in several other individuals based in New Zealand and Pacific Islands who had undergone genetic testing for various diseases. This highlights the need to consider the under-representation of different ancestries in databases that are commonly used as references for interpreting genetic variants identified from NGS, and proceeding to diagnosis.
3. With the increased and frequent use of gene panels for genetic diagnosis, it is highly probable that additional individuals – especially from Pacific regions - will be identified harbouring this homozygous variant in *DOCK8*, irrespective of their clinical presentation. Our findings provide evidence, and assurances, to clinicians and genetic counsellors that this variant is not pathogenic and does need to be considered further in management of such individuals.

## Conclusions

Our study identifies a novel patient homozygous for the previously reported *IFNAR1* c.1156G > T variant found to be common in people of Polynesian ancestry [15]. The clinical features of this case confirms the critical role of type 1 IFN in host defence against some viruses. In hindsight, treatment of SARS-CoV2-induced pneumonia in P1 - even prior to a genetic diagnosis - likely facilitated relatively early recovery and survival given the well-known severity of SARS-CoV2 infection in IFNAR1/IFNAR2-deficient patients [4, 5, 8, 10, 14]. From a clinical standpoint, the detailed genomic characterisation of P1 established the pathogenic role of homozygous *IFNAR1* variants while concomitantly excluding DOCK8 deficiency. This allowed us to rapidly procure the SNP genotyping technology using the dried blood spot card to assess the *IFNAR1* c1156G > T variant in P1’s new born sibling [45]. This infant was found to be heterozygous and thus unaffected (II.3, Fig. 1A). Routine care and vaccination recommendations could then be promptly provided. More importantly, our findings provide a salient example of the fundamental requirement to functionally validate VUS or novel variants with mixed predictions of pathogenicity. This is particularly pertinent when assessing variants found in populations under-represented in large public databases – and thus appear to be rare but are in fact common (MAF > 1%) in specific ancestries. Furthermore, even when found to be common, unique variants need to be rigorously tested because the variants may be pathogenic (i.e. *IFNAR1*) or likely benign (*DOCK8*).

## Data Availability

No datasets were generated or analysed during the current study.
