# Correction to: A Novel Case of *IFNAR1* Deficiency Identified a Common Canonical Splice Site Variant in *DOCK8* in Western Polynesia: The Importance of Validating Variants of Unknown Significance in Under-Represented Ancestries

**DOI:** 10.1007/s10875-024-01801-x

**Published:** 2024-09-19

**Authors:** Aimee Huynh, Paul E Gray, Anna Sullivan, Joseph Mackie, Antoine Guerin, Geetha Rao, Karrnan Pathmanandavel, Erika Della Mina, Georgina Hollway, Matthew Hobbs, Karen Enthoven, Patrick O’Young, Sam McManus, Luke H. Wainwright, Megan Higgins, Fallon Noon, Melanie Wong, Paul Bastard, Qian Zhang, Jean-Laurent Casanova, Kuang-Chih Hsiao, Alberto Pinzon-Charry, Cindy S Ma, Stuart G. Tangye

**Affiliations:** 1 Queensland Paediatric Immunology and Allergy Service, Children’s Health Queensland, Brisbane, QLD 4101 Australia; 2 Clinical Immunogenomics Research Consortium, Australasia, Australia; 3 School Medicine, Western Sydney University, Penrith, NSW Australia; 4 Department of Immunology and Infectious Diseases, Sydney Children’s Hospital, Randwick, Australia; 5 Garvan Institute of Medical Research, 384 Victoria St, Darlinghurst, NSW 2010 Australia; 6 School of Clinical Medicine, Faculty of Medicine and Health, UNSW Sydney, Sydney, NSW Australia; 7 Royal Brisbane and Women’s Hospital, Herston, QLD Australia; 8 Genetic Health Queensland, Brisbane, Australia; 9 Department of Allergy and Immunology, The Children’s Hospital at Westmead, Sydney, NSW Australia; 10 Laboratory of Human Genetics of Infectious Diseases, Necker Branch, INSERM U1163, Necker Hospital for Sick Children, Paris, France; 11 St. Giles Laboratory of Human Genetics of Infectious Diseases, Rockefeller Branch, The Rockefeller University, New York, NY USA; 12 University of Paris, Imagine Institute, Paris, France; 13 Department of Pediatrics, Necker Hospital for Sick Children, AP-HP, Paris, France; 14 Howard Hughes Medical Institute, New York, USA; 15 Starship Child Health, Auckland, New Zealand; 16 Department of Paediatrics, University of Auckland, Auckland, New Zealand; 17 Griffith University and University, Queensland, Australia

**Correction to: Journal of Clinical Immunology (2024) 44:170**

10.1007/s10875-024-01774-x

In this article, Alberto Pinzon-Charry, Cindy S Ma and Stuart G. Tangye should have been denoted as equally contributing authors.

The original version has been corrected.

